# Preparing Medical Specialists for Genomic Medicine: Continuing Education Should Include Opportunities for Experiential Learning

**DOI:** 10.3389/fgene.2020.00151

**Published:** 2020-03-03

**Authors:** Belinda J. McClaren, Erin Crellin, Monika Janinski, Amy E. Nisselle, Larissa Ng, Sylvia A. Metcalfe, Clara L. Gaff

**Affiliations:** ^1^ Australian Genomics Health Alliance, Melbourne, VIC, Australia; ^2^ Genomics in Society, Murdoch Children’s Research Institute, Melbourne, VIC, Australia; ^3^ Department of Paediatrics, The University of Melbourne, VIC, Australia

**Keywords:** genomic education, genomic medicine, medical specialist, workforce, qualitative needs assessment, experiential learning

## Abstract

With the demand for genomic investigations increasing, medical specialists will need to, and are beginning to, practice genomic medicine. The need for medical specialists from diverse specialties to be ready to appropriately practice genomic medicine is widely recognised, but existing studies focus on single specialties or clinical settings. We explored continuing education needs in genomic medicine of a wide range of medical specialists (excluding genetic specialists) from across Australia. Interviews were conducted with 86 medical specialists in Australia from diverse medical specialties. Inductive content analysis categorized participants by career stage and genomics experience. Themes related to education needs were identified through constant comparison and discussion between authors of emerging concepts. Our findings show that participants believe that experiential learning in genomic medicine is necessary to develop the confidence and skills needed for clinical care. The main themes reported are: tailoring of education to the specialty and the individual; peer interactions contextualizes knowledge; experience will aid in developing confidence and skills. In fact, avenues of gaining experience may result in increased engagement with continuing education in genomic medicine as specialists are exposed to relevant applications in their clinical practice. Participants affirmed the need for continuing education in genomic medicine but identified that it would need to be tailored to the specialty and the individual: one size does not fit all, so a multifaceted approached is needed. Participants infrequently attended formal continuing education in genomic medicine. More commonly, they reported experiential learning by observation, case-review or interacting with a “genomics champion” in their specialty, which contextualized their knowledge. Medical specialists anticipate that genomic medicine will become part of their practice which could lessen demand on the specialist genetic workforce. They expect to look to experts within their own medical specialty who have gained genomics expertise for specific and contextualized support as they develop the skills and confidence to practice genomic medicine. These findings highlight the need to include opportunities for experiential learning in continuing education. Concepts identified in these interviews can be tested with a larger sample of medical specialists to ascertain representativeness.

## Introduction

The emerging practice of genomic medicine, the use of genomic information to guide diagnostic and treatment decisions, promises to transform the way medicine is practiced (Collins and McKusick, 2001; Williams, 2019). Yet challenges remain in maximizing the potential benefits within healthcare settings and beyond specialist genetic services (Ginsburg, 2014; Gaff et al., 2017). Zebrowski et al. recently evaluated perspectives on implementing genomic medicine within the IGNITE network (Implementing GeNomics In pracTiCe). While participants identified clinician engagement as essential for genomic medicine implementation, researchers actually observed a lack of clinician engagement among participants studied (Zebrowski et al., 2019). Medical specialists who are not already engaged in providing genetic services will need to “develop and expand” their expertise in inherited diseases and the use of new genomic technologies in their clinical practice (Burton, 2011; Burton et al., 2017; Gaff et al., 2017).

Changes to medical education and training curricula will address this gap over time, but there is a pressing need for those already in practice to be ready to integrate testing and application of test results into medical care. The challenges for medical specialists to integrate genomic medicine into their clinical practice have only been investigated in a piecemeal approach so far, with most studies involving hospital-based specialists from the same specialty. For example, in studies involving oncologists, clinicians reported feeling underprepared to comprehend and communicate genomic test results despite practicing in areas in which the clinical utility of genomic investigations for some conditions or some patients was established and testing was available (Chow-White et al., 2017; Johnson et al., 2017; Weipert et al., 2018). While expressing familiarity with discussing genetic information, cardiologists in the MedSeq study similarly felt underprepared to navigate complex genomic test results, particularly those that lay outside their specialty (Christensen et al., 2016).

**Figure 1:**
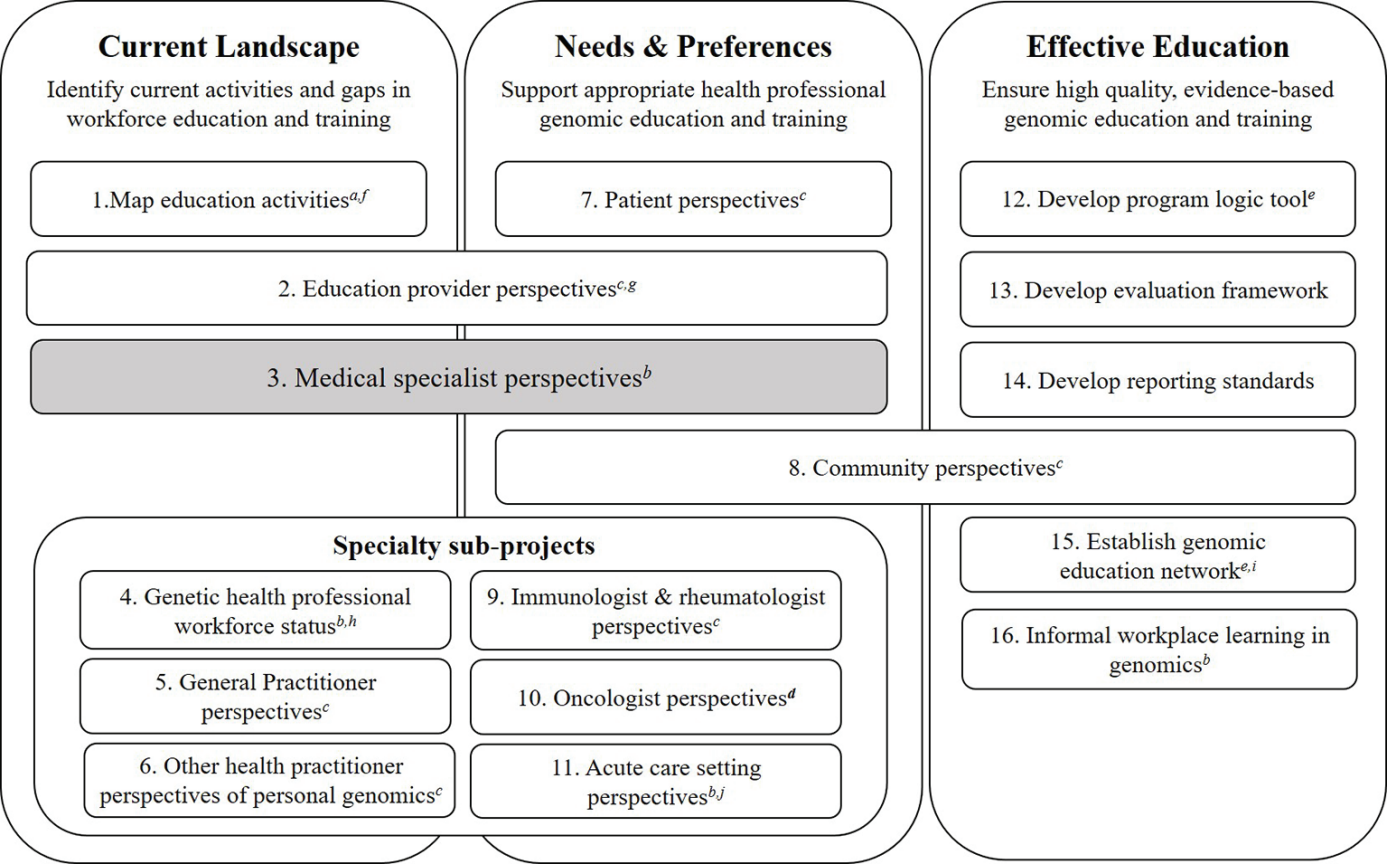
Australian Genomics Health Alliance: Workforce & Education research program design. The Workforce & Education program of Australian Genomics seeks to identify gaps and opportunities around continuing education of health professionals to support the practice of genomic medicine. To achieve this, our research program has three work streams around education and clinical practice: mapping the current landscape; identifying needs and future preferences; and ensuring effective education through evaluation. The present study is shown in grey and has only included medical specialists, defined as “doctors specialized in a field other than general/family practice or clinical/medical genetics” (Crellin et al., 2019, pg 1–2). The data collection methods used in the program are: *^a^*desktop audit; *^b^*mixed methods (qualitative and quantitative); *^c^*qualitative interviews; *^d^*quantitative survey; *^e^*workshop/meeting. Outputs from the program to date are: *^e^*(Nisselle et al. 2019b) *^f^*(McClaren et al., 2018); *^g^*(Janinski et al., 2018); *^h^*(Nisselle et al., 2019a); *^i^*First meeting held August 2018, Sydney; *^j^*(Stark et al., 2019b). Participants groups represented in the studies within this program of research are: medical specialists (3, 9–16), genetic counselors (4, 11–15), clinical geneticists (2, 4, 11–15), bioinformaticians and medical scientists (11–15), genomic education providers (2, 12–15), general practitioners (5, 6, 12–15), patients or parents of patients (7, 15), system influencers and policy makers (8), oncologists (10), community practitioners (pharmacists, nutritionists, private practice genetic counselors (6).

In the U.S.A., a nation-wide study of pharmacogenomics has been conducted (Stanek et al., 2012), but we found no other, nation-wide studies that include a broad range of medical specialties to explore readiness to practice genomic medicine and the role continuing education plays. Yet, the need for education to support the implementation of genomic medicine has been recognised internationally by policy makers (Manolio et al., 2013; Bowdin et al., 2016). For instance, the Australian Government recently released a National Health Genomics Policy Framework^1^ which identified “building a skilled workforce that is literate in genomics” (page 3) as a key strategic priority (Australian Government Department of Health, 2017). However, policy statements such as these need education plans to prepare clinicians to practice and explain the role continuing education can play. In England, the National Health Service invested early in a “top–down” approach with centralized administration to equip the workforce to incorporate genomics through a range of education and training initiatives (Turnbull et al., 2018). Australian investment has been made in national research funding to provide evidence for the equitable, effective and sustainable integration of genomic medicine in healthcare through the Australian Genomics Health Alliance (Australian Genomics) (Long et al., 2019). Australian Genomics is a research partnership of clinicians, diagnostic geneticists and researchers from >80 organizations using a co-ordinated nation-wide approach (Stark et al., 2019a). To inform the development and delivery of effective education and training in genomics across the broad health care system and adoption of genomics by numerous medical specialties the Australian Genomics Workforce & Education research program takes a whole-of-nation, “bottom up,” research approach. A mixed-methods design for the research program is being undertaken to examine the perspectives of multiple stakeholder groups (**Figure 1**) (Creswell and Plano Clark, 2017; McClaren et al., 2020).

We report here the qualitative findings of this nation-wide study of medical specialists, the first to address diverse specialties and career stages. This study has informed the development of a nation-wide quantitative survey capturing representative data across medical specialties and healthcare settings. Specifically, the purpose of this study was to understand how medical specialists in Australia perceive the relevance of genomics to their practice as well as their views on continuing education in genomics to enhance clinician readiness. This manuscript presents findings related to medical specialists’ needs for continuing education in genomic medicine.

## Materials and Methods

Key informant qualitative interviews were conducted and the semi-structured interview guide addressed: participant characteristics; current role; experience with genomic medicine; participation in or attendance at education and training activities; and perceptions of future need for continuing education. This study had human research ethics approval (University of Melbourne, HREC: 1646785). As per the approved research protocol and in accordance with the National Statement on Ethical Conduct in Human Research (Section 2.2.5)^2^, interview participants gave verbal consent for interviews to be audio recorded, transcribed and for de-identified quotes to be used in publications or reports arising from the research. Purposive and snowball approaches were used for maximum variation sampling in order to gather data representative of various genomics experience levels and career stages (Patton, 2015). This included direct email invitation to individuals who have a medical degree and specialist training. The term “medical specialists” is used in this study to mean “doctors specialized in a field other than general/family practice or clinical/medical genetics” (Crellin et al., 2019, pg 1–2). We have separate studies (**Figure 1**) underway or completed with clinical (or medical) geneticists (Nisselle et al., 2019a) and general practitioners (GPs) as we anticipated that the needs of those who are specialized in genetics or those in primary care (i.e. GPs) may be quite distinct and therefore require separate consideration. In Australia, GPs have a different training pathway^3^ to physicians (medical specialists) and their role is typically to refer patients with likely medical conditions to medical specialists or genetic specialists who will examine, investigate and deliver results to patients. Therefore, GPs may need broad knowledge about appropriately identifying and referring their patients who have further need of follow-up, whereas the role of medical specialists is to request diagnostic tests, interpret results, and deliver results to patients. Hence GPs were excluded from this set of interviews.

Interviews were conducted by telephone or face-to-face, and audio-recorded. Recordings were transcribed verbatim, checked for accuracy, and NVivo 12^3^ used to manage qualitative analysis. Participants were stratified using content analysis to explore how their views might differ across genomics experience levels and career stages (Hsieh and Shannon, 2005; Patton, 2015). By inductively analyzing manifest content (self-reported current practice and genomics experience), participants were classified as belonging to one of three genomics experience levels (**Table 1**):

- Novice: no (or rare) use of genomics in clinical practice and/or; no involvement in genomics research and/or; ambivalence towards continuing education in genomic medicine
- Interested: infrequent use of genomics in clinical practice and/or; some (or rare) involvement in genomics research and/or; interest in, but perhaps not attendance at, continuing education in genomics
- Experienced: current use of genomics in clinical practice and/or; active involvement in genomics research (molecular or clinical) and/or; participation in continuing education in genomics.

**Table 1:** Participant characteristics.

Characteristic		N = 86^a^ (%)
**Career stage**	Early (pre-fellowship; junior medical officer)	14 (16)
	Mid (specialist consultant; senior medical officer)	31 (36)
	Senior (head of department; professor)	41 (48)
**Genomics experience**	Novice	29 (34)
	Interested	34 (39)
	Experienced	23 (27)
**Clinical load**	Mostly clinical (> 50%)	36 (42)
	Some clinical (≤50%)	40 (46)
	No current clinical load	10 (12)
**Patient type**	Adult patients only	51 (59)
	Pediatric or obstetric patients^b^	35 (41)
**Practice setting**	Public (hospital or pathology laboratory)	70 (81)
	Private practice only	6 (7)
	Research institute or academic	10 (12)
**Involvement in genomic research****^c^**	Very involved	17 (20)
	Some involvement	36 (42)
	No involvement	33 (38)
**Involvement in education of peers****^d^** **^,^** **^e^**	Very involved	19 (30)
	Some involvement	26 (42)
	No involvement	18 (28)
**Location within Australia**	Victoria & Tasmania	37 (43)
	New South Wales & Australian Capital Territory	19 (22)
	Queensland	19 (22)
	Western Australia & South Australia	11 (13)

a 20 medical specialties were approached with responses from 18: anesthesiology (n = 1), cardiology (n = 1), dermatology (n = 1), endocrinology (n = 4), fetal medicine (n = 2), general medicine (n = 1), hematology (n = 6), immunology (n = 17), infectious disease (n = 2), intensive care (n = 7), nephrology (n = 5), neurology (n = 5), neuropsychiatry (n = 4), obstetrics & gynaecology (n = 2), oncology (n = 6), general pediatrics (n = 4), pathology (n = 8) and rheumatology (n = 10). There were two further specialties approached but no response was received and therefore no interview could be completed: emergency medicine and ophthalmology.

b may also see adult patients.

c Some involvement = Listed on grants, referring patients into research studies, but not running studies themselves; Very involved = Leads research programs (gene discovery, testing patients), holds grants, doing PhD related to genomics.

d Includes having any role in delivering peer education (not just genomic). Some=gives occasional talks to department, sought out by peers for information; Very=organizes and delivers education to peers, recognized as a genomic leader in their field. Of those who do educate their peers, only two have formal background/qualifications in education.

e Data only collected for 63/86 participants due to difference in data collection tools used for immunologists and rheumatologists who were not asked about involvement in educating peers.

Participants were additionally categorized (**Table 1**) into their career stage according to the medical training pathways in Australia^4^:

- Early—junior medical officer who is in their pre-fellowship training years which includes being an intern or a registrar
- Mid—specialist consultant or a senior medical officer who is completing their fellowship training
- Senior—representing medical specialists who are heads of department or who are professorial fellows

A coding framework was developed based on the broad topics from the interview guide with further codes added in an inductive process. The analysis approach was iterative and involved reading and re-reading the transcripts using constant comparison to identify similarities and differences, and discussion between coders (BM, EC, MJ, LN) of emerging concepts (Vaismoradi et al., 2013; Patton, 2015). All the transcripts were coded once and the full codebook developed. All transcripts were then coded a second time using the codebook. Regular discussions between all four coders managed the development of codes, the emergent concepts and helped resolve conflicts among coders.

## Results

From January 2017 and May 2018, 240 medical specialists were invited to participate in the study. Interviews were conducted with 86 medical specialists from 18 different specialties (**Table 1**). Interviews were held with all who responded and for whom an interview could be arranged, which allowed for an inclusive approach with broad representation of a variety of participants. Findings are shown below using representative quotes as exemplars and attributed to participants using study numbers and descriptors of their specialty. Some quotes have been truncated for readability without changing the meaning, indicated by “…”.

**Figure 2:**
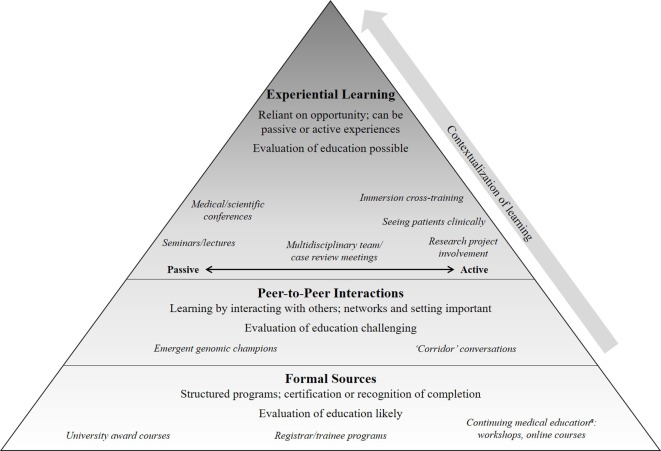
A summary of the participant-described approaches to education and learning that can prepare a medical specialist to practice genomic medicine. Formal sources of education, such as structured programs, provide knowledge that is then contextualized through peer-to-peer interactions and opportunities for experiential learning; each of these can build upon each other although are not necessarily equal in quality and quantity. Defining preparedness is challenging and may vary for different types of specialists (Vassy et al., 2015); we use this term to encompass knowledge, attitude, skills and confidence (Crellin et al., 2019). *^a^*These activities are ones in which medical specialists would receive recognition from their relevant medical College, such as “points,” for having completed the educational activity.

All participants affirmed the need for continuing education in genomic medicine. Findings from participants related to continuing education for medical specialists are presented and summarized into the following themes: tailoring of education to the specialty and the individual; peer interactions contextualizes knowledge; experience will aid in developing confidence and skills. The concepts covered in the sections below are interlinked due to the nature of how the participants spoke about their interactions with genomic medicine and their needs for continuing education. While presented in separate sections, the illustrative quotes may convey more than one idea from more than one section. **Figure 2** provides an overall conceptual representation of the emergent concepts: showing how a foundation of knowledge from formal sources is built on by interactions with peers to begin to contextualize knowledge. As medical specialists have opportunity to gain experience in genomic medicine, their confidence and skills grow. As well as the three themes, we present challenges to continuing education identified by the participants.

### Continuing Education in Genomic Medicine Needs to be Tailored to the Specialty and the Individual

Medical specialists identified that one size does not fit all for approaches to continuing education in genomic medicine. Participants having experience with genomics, and therefore a greater level of confidence to practice genomic medicine, had different needs for continuing education compared with those who were less experienced.

*“For me, (current education activities are) fairly good and adequate, I already come into it with a knowledge of genomics (through involvement in genomics research). I’m not sure for the general clinician whether there is enough opportunities … to upskill them.” [MS23, senior, experienced, neurologist]*

*“Education needs … it’d be a few tiers of education, so a general education to the general health service providers, as well as a targeted, more in-depth education to those who (currently use genomics).” [MS53, senior, interested, nephrologist]*

Genomics novices sought out basic information and updates, whereas others with more experience wanted greater detail.

*“…if it goes into a lot of detail, I just start to get confused and tune out a little bit … I’m interested to have a bit of an idea about how it works.” [MS54, early, novice, pediatrician]*

*“(What) I need is a refresher course with a very clinical tilt to it. I do not want to know the A T G C, but I want to know, when you say genome exome sequencing or whole genome sequencing, what do you do, what are the results you get, and how do you make those decisions after that, as in how do you report them.” [MS57, mid, interested, intensivist]*

Participants also discussed how career stage might influence how they would like to learn about genomic medicine.

*“Registrars would probably be quite happy doing (webinars)…the older people get, the less inclined they probably are to engage in that way … there is a limit to how much you can actually get from sitting there looking at the video.” [MS21, senior, experienced, immunologist]*

There were conflicting views regarding baseline understanding of genomics concepts: some thought recent medical graduates would have more knowledge, gained in their formal education than senior physicians, yet in contrast others described that current medical school and training curricula appeared to have limited genomics content.

*“Someone who graduated from medicine in 2014 is going to have a very different baseline knowledge of genomics than someone like me who … finished the medical course in (196-).” [MS19, senior, novice, immunologist]*

*“I’m still surprised how little genetics the current trainees know … the actual training to be a physician or a sub-specialist, there still seems to be very little formal genetics training within that.” [MS23, senior, experienced, neurologist]*

*We didn’t do a lot (in medical training)…most people came (to medicine) from basic biomedical degrees…(In medical training) there’d be a mention of something in a lecture on pediatric cardiology about various different genetic conditions that you have for pediatric cardiac genetic mutations … We didn’t, in medicine, go through the molecular basis of how that happens. [MS30, early, novice, general medicine physician]*

A common theme from all participants was the importance for continuing education to be clinically relevant and tailored to the audience. Clinicians wanted the pitch and scope of information to be tailored to their specialty, and relevant to the patients they see now or anticipate seeing in the near future.

*“The different specialties may be very different … if you’re thinking about oncology genetics, the relevance for neurology would be very different.” [MS27, senior, novice, neurologist]*

*“(A) seminar or some sort of update … put within a context that clinicians would recognise it as being directly relevant to what they do day-to-day, rather than relevant to them in 10 or 15 years’ time.” [MS32, mid, interested, rheumatologist]*

To further identify continuing education needs, participants were also asked to suggest topics to be addressed for continuing education in genomic medicine to support their readiness to practice genomic medicine (**Table 2**). A spectrum of content was described, from basic, to practical, to technical and clinically applied/or advanced (e.g., the precise phenotypic information required to make decisions about gene lists or variant classification). Participants also identified other skills, such as communication and counseling, for example, helping families understand implications of genomic data storage and use, and interpretation of detected variants.

*“(I’d like to learn about) the technology itself, the limitations, the patient selection, the counseling around the results and the meaning of the results and how you work through the variants that you’re not sure of.” [MS27, senior, novice, neurologist]*

**Table 2:** Topics suggested by participants for continuing education to support their readiness to practice genomic medicine.

Sub-category	Representative quotes
**Threshold concepts**	*“Genomics 101…it seems to be advancing fast.” [MS31, senior, novice, rheumatologist]*
**Language and terminology**	*“The major barrier has been language … challenging to keep up.” [MS03, senior, interested, endocrinologist]*
**Limitations of genomic approaches**	*“You have to know what the limitations of the test are … and the limitations of the bioinformatics process that you’re using.” [MS26, senior, novice, fetal medicine specialist]*
**Guidelines and resources**	*“We need to know where to go to get the information … what websites, what resources, and who are the contact points locally or nationally or internationally?” [MS36, mid, interested, nephrologist]*
**Creating gene lists**	*“I’d like to know how they create the (gene) list of interest.” [MS33, mid, interested, neurologist]*
**Documenting and communicating relevant phenotypic information with test requests**	*“One of the first things I will do is examine from top to toe. There are some physical features that we might not flag or have the right language for … I’m constantly seeing the geneticist then put their phenotype description down and there are some things in there that are new or I don’t recall to mind as often.” [MS46, mid, interested, pediatrician]*

### Learning From Peers Contextualizes Knowledge in Genomic Medicine

Participants described how interactions with peers contextualizes formal learning in genomic medicine, which required participants to have peer networks they could draw on, physically, if the setting allowed it, or by phone.

*“I can walk down the corridor and talk to (a clinical geneticist).” [MS04, senior, interested, endocrinologist]*

*“We’re very spoiled here … pick up a phone or … get the geneticist to see them. It might be different for pediatricians out in the rest of the world.” [MS54, early, novice, pediatrician]*

Some peers were identified as particularly useful: these were described by participants as “champions,” who are medical specialists, usually within the same medical specialty, with a special interest in genomics and would readily share their genomics expertise with others.

*“I think one of the hopes is that I will be a little bit of a link and help upskill…(using) my learning … spread a little bit of that (to my colleagues).” [MS47, early, experienced, neuropsychiatrist]*

*“Find a few people who are in the intensive care (ICU) scenario who are your champions, and have the ICU guys talk to the ICU guys … rather than have Genetics coming in, giving a talk and intensivists only understanding half of it.” [MS63, senior, experienced, intensivist]*

*“Maybe keep it just in the hands of the few competent people in every specialty who can handle this and who can advise others about what the consequence of certain findings are.” [MS52, senior, interested, nephrologist]*

### Opportunities to Gain Experience in Genomic Medicine Promotes Confidence and Skills

Few participants were aware of or had attended any formal continuing education courses or workshops in genomic medicine as shown in **Figure 2**. Participants described addressing these needs instead through experiential learning opportunities in the following ways: passive approaches such as attendance at conferences or seminars; active learning through research projects, seeing patients in clinical practice, or undertaking immersive cross-training; or multidisciplinary team (MDT) meetings where clinical cases are reviewed, which could be a combination of passive and/or active learning. Representative quotes of the ways in which participants identified learning through opportunities to gain experience are summarised in **Table 3**. Specifically, participants described how MDT meetings gave them the opportunity to learn passively by hearing cases of their peers, and also to be active contributors by nominating their own cases and taking part in discussions around gene list prioritization or variant classification.

**Table 3:** Participant descriptions of approaches to learning to support practice of genomic medicine.

Sub-category		Representative quotes
**Passive learning**	Conference attendance	*“(named) conference which has quite a lot of genetics as part of its presentations and education sessions as well.” [MS11, early, interested, hematologist]*
	Department meetings	*“When members of our team go (to conferences) we discuss them all together, discuss breakthroughs in the literature on a weekly basis.” [MS05, senior, experienced, endocrinologist]*
**Active learning**	Involvement in research	*“While I have ordered some genomic tests and given some results, I’ve done a lot more research.…a bit of learning by osmosis … so informal things.” [MS13, mid, interested, neurologist]*
	Seeing patients	*“Really it’s (understanding of genomic medicine) increasing purely by discussing cases, seeing patients.” [MS53, senior, interested, nephrologist]*
	Immersive	*“I’ve got sabbatical time in my contract and study leave. I think it’d (immersive training) be worthwhile, only take a week or two weeks off or whatever to get, immersed in it, into it all.” [MS53, senior, interested, nephrologist]*
	Teaching others	*“I give lectures … so I had to read up … to present it to everyone. So I think there is lots and lots of self-education.” [MS15, senior, experienced, hematologist]*
**Combination learning**	MDT^a^ meetings	*“We talk about difficult clinical cases and we get (genetic) specialist (involved).” [MS54, early, novice pediatrician]*

a Multidisciplinary team.

*“…multidisciplinary meeting with experts from different areas present in the room to assist in making management decisions about patients … As a learning exercise for clinicians it was incredibly valuable to be … benefiting from the expertise of scientists.” [MS99, senior, interested, oncologist]*

Opportunities to learn by gaining experience were variable for participants in this sample. For most participants, such experiential learning was possible due to genomic medicine increasingly becoming part of their clinical practice or likely to be in the near future. Participants recognised that some fields would have more opportunities for experiential learning compared with others because genomic medicine was more relevant and available.

*“The microbiologists, the hematologists, the geneticists and the endocrinologists were all very early adopters of genomics because of the sort of conditions we see and the ease of sample collection. [MS03, senior, interested, endocrinologist]*

Others described their limited experience with exome testing and that their current approach would be to refer to a geneticist and therefore they are not gaining experience themselves. Without these opportunities to learn and limited (to date) experience in delivering genomic medicine some participants felt they were less confident to practice.

*“I have referred patients to geneticists with the specific question of ‘is this patient suitable for exome sequencing?’, but I haven’t actually put in an order for it myself.” [MS54, early, novice, pediatrician]*

*“I certainly wouldn’t feel comfortable looking at reports myself, and relying on my own interpretation. I would think I’d need many more years of looking at that before I’d be comfortable.” [MS11, early, interested, hematologist]*

### Challenges to Learning Identified

Preferences for learning were asked of all participants and although formal continuing education activities such as workshops, short courses and online courses were raised, the following quote exemplifies the decisions participants made about the benefits and competing demands in attending education sessions.

*“Can I physically attend this? Is it possible given my shift schedule, and then is this a skill I either want to get better at or I really need?” [MS30, early, novice, general medicine physician]*

While learning through peer-to-peer interaction or experiential learning, were commonly-mentioned means of developing skills and confidence in genomic medicine, participants did not view these as “education” *per se*.

*“It was just kind of *ad hoc*, learning as you go … I did spend some time in the molecular genetics lab … I did go to curation meetings … It worked for me, except it wasn’t formal teaching where you actually get through the patients being presented, it was more, picking up and asking little questions here and there about very basic things. But it wasn’t structured education or anything.” [MS33, mid, interested, neurologist]*

Participants described how experiential learning was also not equally available across different settings, for example less so in the private sector, or where genomic medicine was infrequently practiced. This was considered a barrier for some medical specialists to upskill in genomic medicine.

*“It’s hard in the private sector … in the public sector you have MDTs. We don’t have much of that so I think that’s where it’s lacking.” [MS25, mid, experienced, hematologist]*

## Discussion

This study provides new insights applicable to meeting the continuing education needs in genomic medicine of diverse medical specialists. The need for education in response to increasing availability of genomic testing in clinical settings has been previously demonstrated (Manolio et al., 2013; Bowdin et al., 2016; Burton et al., 2017; Paul et al., 2018). We extend findings from earlier studies of select medical specialists with this cohesive study exploring a large national sample with diverse specialties, career stages, public and private practice settings and (in)experience with genomics.

Our findings show that motivations to engage with continuing education about genomic medicine appear to be driven by a combination of: individual characteristics (interest in genomics, career stage, and medical specialty); perceptions of relevance to practice (current and future); and prior experience, such as that gained in research settings. We have shown that medical specialists contextualize their knowledge gained through formal education by engaging with their peers and seeking out opportunities for experiential learning. In fact, participants described how most genomics learning occurs outside of attendance at continuing education activities, which have been the previous focus of workforce development (Burton, 2011; Talwar et al., 2017).

### Continuing Education Activities Should Include Opportunities for Experiential Learning

Experiential learning approaches are consistent with adult learning theory, which acknowledges the role of experience and relevance to work settings. Encountering clinical problems will be drivers for medical specialists to self-identify areas of education need and will motivate them to participate in activities to fulfil the gaps in competence or confidence (Grant, 2002; Metcalfe et al., 2008; Knowles et al., 2015). Opportunities for experiential learning should be provided alongside formal continuing medical education activities in genomics. Despite the calls for formal education programs for health professionals in genomics (Passamani, 2013; McGrath and Ghersi, 2016), the medical model of structured “bedside” teaching would also be an appropriate approach for integrating the skills to practice genomic medicine in real-life contexts (Peters and Ten Cate, 2014).

Learning, as described by participants in our study, may include a gradual building of experience, confidence and procedural skills that are specific to the way a specialist may practice genomic medicine. Learning in this context was described as most likely to come from their colleagues who were more experienced in genomics. Such people need to be fostered in their roles as “genomics champions” within their specialty to ensure they demonstrate appropriate competence and are given time and support to teach others. This collegial learning may be less accessible in more isolated sites, such as private practice and more geographically remote settings, so attempts to re-create these opportunities are needed, perhaps by teleconference or telemedicine. Future research is needed to assess the acceptability and feasibility of this approach. Telemedicine in oncology settings has been used effectively to convene virtual tumour boards and educate clinicians (Satcher et al., 2014).

### The Complexity of Providing Continuing Education in Genomic Medicine: The Need for a Multi-Level Approach Across Broad Topics

A nuanced and comprehensive view of learning needs to be taken to ensure medical specialists are equipped to provide genomic medicine to their patients. Specific continuing education activities may provide one approach, but this study suggests that medical specialists will engage more with experiential learning. Such learning may be more likely to encourage medical specialists to adopt genomic medicine when: they feel confident in the clinical utility of genomic medicine; their clinical setting supports genomic testing; and they have developed networks and relationships with colleagues, including those seen to be “genomics champions” within their specialty. As highlighted in a review by Paul et al, evidence for the effectiveness and importance of educational activities is lacking, with current understanding from published studies suggesting many other important domains will contribute to the behaviour change required for the widespread adoption of genomic medicine (Paul et al., 2018).

Clearly, no one size or one time-point for education fits all; therefore, a multi-level approach will be needed to ensure life-long learning is available to support the implementation of genomic medicine into healthcare. Our data suggests this might include efforts to ensure that foundational or threshold concepts of genomic medicine as well as practical skills (terminology, limitations, guidelines, required phenotypic information, and result generation) be included in continuing education (**Table 2**). As shown in **Figure 2**, knowledge of these topics can then be applied and contextualized over the professional life-course of the medical specialist. As the medical specialist encounters genomic medicine in their practice and has a developing sense of its relevance to their patients, they are likely to seek out continuing education to support their practice. Continuing education has the role, therefore, of providing practical examples of genomic medicine to enhance specialists’ confidence and skills to practice.

Our findings also show content areas participants felt would be valuable to address in continuing education (**Table 2**). Regardless of the content topic in focus, relevance to clinical practice is essential for learning, therefore the specialist’s clinical practice influences their perception of relevance of genomic medicine and motivation to undertake continuing education (Burke et al., 2006; Reed et al., 2016). A foundational, baseline understanding of genomic concepts allows a common language to be used and understood in communication, then practical training is needed to convert fundamental understanding into confident practice (Stanek et al., 2012). This common language and understanding would encourage good relationships between scientists and clinicians, which is essential for efficient clinical outcomes (Burton et al., 2017; Weipert et al., 2018).

If learning is occurring predominantly experientially rather than *via* structured activities, evaluation of teaching opportunities will be challenging. Australian Genomics has formed a working party to create a genomics education evaluation framework: international experts in genomics education, evaluation and implementation science met for a workshop in February 2018 to draft a program logic and evaluation framework, which is being refined and tested with member educational activities. A separate publication describes the framework and its development (Nisselle et al., 2019b).

### The Importance of Needs Assessments in Developing Continuing Education Programs

To evaluate the extent of the use of formal education, we previously undertook a mapping exercise of continuing educational activities available to medical specialists in Australia for genomic medicine (McClaren et al., 2018). This mapping and interviews with providers of educational activities demonstrated that most people delivering such education are clinicians rather than educators (Janinski et al., 2018). They may, therefore not think of experiential learning as a strategy to include in the design of their educational activity. The recommendation in this paper to incorporate experiential learning into continuing education activities is aimed at clinicians (and educators) who are currently providing continuing education in genomic medicine for medical specialists. These findings can assist those who are charged with the continuing professional development of a single medical specialty or a hospital- or system-wide program to provide the most acceptable and feasible approaches for medical specialists to learn about genomics.

Our findings can also inform needs assessments ahead of producing continuing education programs in genomic medicine; such an approach has previously led to the development of successful and effective education programs (Gaff et al., 2007; Carroll et al., 2009; Houwink et al., 2011; Houwink et al., 2014; Reed et al., 2016). These qualitative findings have already been used to create a survey tool which can be used in international settings to measure physician preparedness for genomic medicine and their preferences for genomics continuing education (McClaren et al., 2020). When using a program logic model to develop education activities and initiatives, an important component of the planning phase is conducting a needs analysis (Nisselle et al., 2019b). The current study has served as a needs analysis to inform the development of educational activities locally after presentation of findings at a workshop in August 2018 (**Figure 1**, item 15. Establish genomic education network).

### Limitations and Future Directions

Although all health systems have unique features, there is a commonality in the challenge of preparing health professionals for genomic medicine. A strength of this study, the broad sample interviewed, means that findings from our study may have wider relevance and inform local needs assessments. It is a limitation that, despite attempts, not every specialty of medicine is represented so there is further need to seek input from missing specialties. A qualitative approach provides a rich data set to inform future studies to assess the representativeness of our findings; this is underway, with an Australian survey of medical specialists (**Figure 1**) (McClaren et al., 2020). Further, these data are a point-in-time perspective of medical specialists suggesting the need for opportunities for experiential learning, within a largely pre-adoption of routine practice of genomic medicine. It is possible that as genomic medicine is more routinely practiced, the need for experiential learning may lessen.

## Conclusions

In summary, our data suggest that approaches to continuing education in genomic medicine should consider:

- Experiential, hands-on learning opportunities that are closely aligned to how genomic medicine will be delivered in practice
- Integrating learning into clinical practice with emphasis on practical skills as appropriate to the clinical setting
- Leveraging opportunities to learn from peers and professional networks, such as involvement in MDTs
- Fostering “genomics champions,” who can advise colleagues on specialty-specific approaches to genomic medicine.

Ultimately, with the demand for genomic investigations increasing, medical specialists will need to, and are beginning to, practice genomic medicine. Our findings show that medical specialists expect to look to experts in their own medical specialty for specific and contextualized support to competently and confidently practice genomic medicine when appropriate to their clinical need. These findings have been used to create a survey tool which can be used to measure physician preparedness for genomic medicine and preferences for continuing education in a representative sample (McClaren et al., 2020). However, it is clear that continuing education in genomic medicine will need to be multifaceted to meet the diverse needs of medical specialists and should include opportunities for experiential learning.

## Data Availability Statement

The datasets generated for this study are available on request to the corresponding author.

## Ethics Statement

The studies involving human participants were reviewed and approved by University of Melbourne, Human Research Ethics Committee. Written informed consent for participation was not required for this study in accordance with the national legislation and the institutional requirements.

## Author Contributions

BM, AN, SM, and CG conceived the idea for the manuscript, and BM and EC conducted the interviews. BM led the analysis which was also completed by EC, MJ, and LN. BM drafted the manuscript and all authors revised drafts, approved the final version, and agree to be accountable for all aspects of the work.

## Funding

This work was supported by the Victorian Government’s Operational Infrastructure Support Program and a grant from the Australian National Health & Medical Research Council (GNT1113531); the contents are solely the responsibility of the individual authors and do not reflect the views of the NHMRC. EC was supported by the Helen R. Freeman Scholarship.

## Conflict of Interest

CG and SM are co-editors of the Research Topic where this manuscript is featured. All authors share their affiliations with CG and SM. CG and SM were not involved in the editorial or peer-review process; this was overseen by the third co-editor who is not affiliated with the authors’ institutes.

The authors declare that the research was conducted in the absence of any commercial or financial relationships that could be construed as a potential conflict of interest.

## Footnotes

1
http://www.coaghealthcouncil.gov.au/Portals/0/Genomics%20Framework%20WEB_1.PDF

2
https://www.nhmrc.gov.au/about-us/publications/national-statement-ethical-conduct-human-research-2007-updated-2018#toc:296, The National Statement on Ethical Conduct in Human Research (2007) (National Statement (2007) consists of a series of guidelines made in accordance with the National Health and Medical Research Council Act 1992. The National Statement is developed jointly by the National Health and Medical Research Council, the Australian Research Council and Universities Australia.

3 NVivo. (2018). NVivo qualitative data analysis software. 12 ed (QSR International Pty Ltd).

4 (https://ama.com.au/careers/becoming-a-doctor) Doctor Life Cycle.
